# Preparing Sections of Teeth for Histology and Bacteriology

**Published:** 1891-07

**Authors:** Vida A. Latham

**Affiliations:** Ann Arbor, Mich.


### Preparing Sections of Teeth for Histology and Bacteriology.

### Part I.—Histology Practical.

VIDA A. LATHAM, ANN ARBOR, MICH.

Part of a paper with additions read at the Student's Dental Society U. of M.

(Continued from page 281)

### CLASS II.—SOFTENED SECTIONS.

For this purpose several reagents may be used but all seem to act by the removing of the calcarious matter and the hardening of the soft tissues of the teeth. It is most important that the last should be thoroughly well attended to or else we do not get a correct idea of the character and structural size of the elements found in the tooth. Chromic acid is the first, a $\frac{1}{6}$th per cent. up to $\frac{1}{2}$ may be used, the former is preferable and is made by adding one gramme to 600 cc of distilled water or 15 grains to the pint, a quantity of which should be kept on hand in order to immerse a freshly extracted tooth that may be desired for examination. Tie a piece of cotton round a fresh tooth and suspend it in the fluid (1 part of spirit to 2 parts acid stir.) so as to be covered by a quantity of the solution. Where the crown of the tooth is not desired for examination it is better to saw it off at the neck so as to allow the stains and reagents to penetrate the tissues. The hardening fluid must be changed the second day and then the fifth and eighth days, the ninth day it is placed in a mixture consisting of two parts of spirit and one of water well stirred, the tenth day into pure spirit and left till desired to use. If desired to soften at the end of the eighth day a few drops of hydrochloric acid can be added to decalcify, which may be tested by passing a fine needle through the tooth, then wash well to remove the acid, then place for a day into 2 parts spirits and 1 water and the next day into ordinary spirits, on the fourth day embed and cut. To embed in parafin, a small tin or paper mould is made and some melted parafin poured into it, the tooth is quickly dipped in and withdrawn till cool. As the parafin is now cooling, place the tooth in it near the hand of the operator and when set withdraw the needle. Place it in a cool place to harden. To prevent the object becoming displaced it should first be dipped in alcohol for some minutes in order to dry the surface thoroughly, the sections are cut with a razor and kept flooded with alcohol, and the sections floated off the knife into a capsule of the same. If desired, a mixture (by weight) of white wax and olive oil, equal parts, melt them together and pour into the embedding dishes, may be used in place of the parafine if desired.

*To Embed in Gum.* If the tooth is in alcohol, transfer for 6 hours into distilled water, then to a gum solution for 6 hours (made of picked gum arabic dissolved in water). Place a fair quantity of the gum on the plate of the microtome such as Cathcarts or Williams, and cool till nearly frozen. Place the tooth in position and surround with gum and let it freeze under a capsule or by means of the spray till solid but not brittle. The gum should cut like cheese. When the sections are cut transfer with a camel hair pencil from the knife to warm distilled water to dissolve out the gum. From water transfer to spirit and spread the sections out evenly. If the sections are delicate they may be transferred directly to the slide and treated with dilute spirit in situ. These may be washed with water and stained with any agent or gold chloride and subsequently dehydrated and cleared and mounted in any media.

*Decalcified Sections.—Picric Acid.* A saturated solution in distilled water is the safest decalcifying agent and it must be kept saturated by the addition of fresh crystals every few days. When soft enough, prick with a needle to test it and then wash well in clean water to get rid of the acid and then in weak spirit which will dissolve the acid more. Keep in pure alcohol and treat as chromic acid for embedding, cutting, &c. Sections may be double stained with picro-carmine and logwood, &c.

*Muller's Fluid.* This is one of the most useful hardening agents we possess. Bichromate of potash, 2 parts, sulphate of soda 1 part and water 100 parts. Put the salts into a pan and some of the water and boil till all dissolved. Add more of the water to cool it and put in a stoppered bottle to keep. After the first few days it does not require changing, but requires a longer time to harden according to the size of the tissue and is a great advantage, as specimens are in no way harmed for the demonstration of micro-organisms. When the tissue is properly hardened, pour off the hardening agent and wash well with water to get rid of the reagent, transfer to weak spirit (2 parts spirit, 1 water, stir.) for a day or two, then into pure spirit.

*Sections of Pulp.* Crush a newly extracted tooth in a vice or with a hammer, and select several pieces of dentine with portions of pulp adherent to them, then immerse in staining fluid, cover with a glass capsule and leave in a warm place for a couple of days. Then pour the fluid off and wash the specimens in a solution made of strong glycerine 2 parts, distilled water 1 part, and leave for a couple of hours to soak. To enable the tissues to regain their original volume, transfer them to a solution of five drops acetic acid to 1 ounce of strong glycerine, and leave in the fluid for four days.

Another method and one recommended by Dr. Bodecker for preparing pulp tissue is to immediately place the tooth after removal from the mouth in a $\frac{1}{6}$ to a $\frac{1}{2}$ per cent. chromic acid. To this mixture add, after the third or fourth day after decalcification, a few drops of dilute hydrochloric acid. It is important to use a large quantity of the liquid and renew every 6 or 8 days. After the teeth have been hardening and decalcifying for a few weeks, the peripheral portion of the dentine will become sufficiently soft to be cut by a razor. When the hard portions of the dentine are reached by the cutting instruments, the extraction of the lime-salts must again be continued in the manner described above until the pulp cavity is reached. If care is used the tooth, especially the anterior, can be split with a strong pair of excising forceps. Then take a $\frac{1}{2}$ per cent. solution of sodium chloride in distilled water (warm) and place on the pulp and drop it without handling into the stain, carmine, logwood, fuchsin, hyperosmic acid, picro-indigo, or chloride of gold, $\frac{1}{2}$ per cent., etc.

Pulp sections, if thin, after hardening in chromic acid can if first washed in distilled water, be stained with gold chloride, leaving the stain on the tissue for 20 to 30 minutes, when they should again be washed in distilled water and exposed to daylight. In a few hours the color of the fresh tissue changes to a bright violet color, whilst the chromic acid pulp is of a brownish violet. Osmic acid, 1 per cent. shows the contours of the constituent tissues, more especially are the nerve fibres distinct. Again both fresh and chromic acid specimens may be treated with osmic acid. Carmine is, perhaps, the best stain for pulps.

To examine the pulp, together with the enclosing dentine, the specimen is softened in chromic acid and then imbedded in celloidin of parafin. or wax, as above described. The fresh pulps of lower incisors are the thinest and best adapted for examining the system of blood vessels and if transferred to the slide when fresh, add some normal saline solution, cover and with careful pressure the pulps may be spread and examined.

If desired, to study *The Blood-Vessels of the Pulp*, chloroform an animal and just before respiration ceases, open the right auricle and let the vessels empty themselves; then inject with Prussian blue, warmed to a temperature of 40° C., to render the gelatine fluid and also to prevent any vascular spasm which a cold fluid is very liable to produce. Then place the head in alcohol for 24 hours to harden the injection; or if preferred in Muller's Fluid or chromic acid which is just as good and in my opinion better. The pulp, after hardening in alcohol is removed and immersed in a weak solution of chromic acid, and at the end of ten days sections of it may readily be cut and then mounted in glycerine jelly. If the animal is quite dead, you must wait till rigor mortis has passed off and inject a non-gelatinous Prussian blue, but the first injection is the best. In animals which die of strangulation the vessels will be found so gorged with blood as to render any further preparation unnecessary.

If the tissues are partially decalcified in a very weak solution of chromic acid and treated as above described, sections can be frozen, cut, stained and mounted so as to show the dentinal fibrillæ as prolongations of the odontoblasts.

I prefer to harden teeth well in Muller's then spirit and then grind sections keeping *them wet* all the time and if wished they can, after grinding, be embedded in celloidin and decalcified in $\frac{1}{2}$ per cent. solution of chromic acid, then treated and stained as desired. A point worthy of remembrance is the dissimilarity between caries of bone and decay of teeth as the reaction is totally different when they are treated with picro-carmine.

*Perenyi's Fluid.* As yet I have had very little chance to thoroughly test this agent to which Dr. N. S. Hoff, of the University of Michigan, very kindly drew my attention, but it seems as if it will be a good agent, having the properties of hardening and decalcifying at the same time. The formula is, nitric acid (10 per cent.) 4 parts, alcohol 3 parts and chromic acid (0.5 per cent.) 3 parts. Mix. Leave the tooth in this agent some 4 hours or more until soft, which is facilitated if the tooth can be cut through at the neck so the agent can enter the canal. Then transfer to 70 per cent. alcohol for 24 hours, strong alcohol for some days, absolute alcohol 4 – 5 days. For ordinary tissues combine the stain with the fixing fluid. Fuchsin may be dissolved directly in the fixing solution. But eosin, purpwein, aniline violet must first be dissolved in 3 parts of alcohol, and then shaken into the liquid.

Picro carmine and borax-carmine can also be added but as a precipitate results it must be filtered and after staining pass through 50 per cent. alcohol for 5 hours, ordinary spirit 10 hours, and then into absolute alcohol. If the tissue is unstained it may, after cutting, be immersed in clove oil colored with alcoholic solution of eosin or sapranin, or on the slide for 5 to 10 minutes, with a few drops of the colored oil.

Kleinenberg's fluid is also useful for decalcifying teeth.

*To Clean Cover-Glasses.* Take from the wool and place in a beaker, cover with strong sulphuric acid, stir to avoid all the air remaining amongst them and leave for an hour or two. Then wash WELL in *several* changes of water to get rid of the acid, and place in alcohol from which they are taken one by one and wiped dry on an old handkerchief and then polished with a chamois leather and kept for use in an air-tight box.

*Retaining the Soft Parts of Bone and Teeth.* Take a fresh tooth or nearly fresh, divide *at once* with a sharp fret saw below the neck into two or three pieces "allowing distilled water to trickle over it the while," and then the reagents and stains can penetrate the pulp cavity. Place the pieces in concentrated sublimate sol. for some hours to fix the soft parts. Wash in running water for about 1 hour, then place for 12 hours in 30 per cent. spirit, change to 50 per cent., and again after a similar period to 70 per cent. To remove the black sublimate precipitate place the teeth for 12 hours in 90 per cent. spirit to which 1.5 to2.0 per cent. tincture of iodine has been added. The iodine is afterwards removed by immersion in absolute alcohol until the teeth become white.

*To Stain in Borax-Carmine* either an alcohol or aqueous sol. gives the best results.

Remove the teeth from absolute alcohol to running water for 15 to 30 minutes and then place in the stain. In the watery solution of borax carmine they remain 1 or 2 days, and in the spirituous 2 or 3 days. Transfer to acidulated 70 per cent. spirit (70 per cent. spirit 100 cen. acid muriat 1.0) in which they remain, the watery ones stained require, at least 12, and the alcohol stained ones 24 to 36 hours. This done, immerse for about 15 minutes in 90 per cent. spirit and then for half an hour in absolute alcohol, after which they are transferred to some ethereal oil for 12 or more hours, then quickly wash the ethereal oil off the objects with pure xylol, and then they are passed in a solution of balsam in chloroform. The balsam (Weil 6 & 2) is prepared by drying in a water bath heated gradually up to 90° for 8 hours or more, until when cold, the mass will crack like glass on being punctured. Of this balsam so much is added to the chloroform as to make a thin solution in which, as stated above, the teeth must lie for 24 hours. Then add as much balsam to the solution as will dissolve. When no more balsam will dissolve, the teeth and a sufficiency of the balsam are poured into a vessel and heated up to 90° in a water bath until the mass, when cold, shall be as hard as glass. Let the balsam set; pick out the teeth carefully, place in a vice, and thin discs are cut from them with a fret saw, water being trickled over them the while. Mount sections in balsam chloroform.

*Teeth to Show Odontoblasts in situ.* Take the jaw, preferably the lower of an embryonic mammal such as a kitten or pup, and while still fresh carefully stripped of the tissues covering it except the oral epithelium and flange of gum, and place in the usual Muller solution 20 to 30 times in volume to bulk of the immersed tissue. Change the fluid every day for 4 or 5 days, and then every 3d or 4th. Then finish hardening after it has been in Muller's fluid for a fortnight—in spirit first weak, then strong, changing till no more color is seen. Vertical sections are cut by a thin sharp knife; place longitudinally on the stage of a Cathcart or William's freezing microtome and cut in the usual way. The best specimens are got in the canine and bicuspid regions for these parts are less likely to be disturbed in the manipulation processes. Imbedding in wax or paraffine or celloidin is of little service. The knife cuts quite easily the thin cap of semi-calcified dentine and bone and the elements of the pulp are in no way disturbed in their relation to each other. The odontoblasts can be separated if necessary, by separating with the point of a needle from the surface of the dentine papilla, the cap of dentine to which, in places, they adhere. (c) This method affects little, if at all, the relative positions of dentine, odontoblasts and pulp.

*To Make Preparations of the Teeth of Fishes in Situ* (8) it is best not to grind down sections of the teeth but to decalcify the jaw and teeth with a 5 per cent. solution of $H_2Cro_4$ or 10 per cent. sol. of HCl. Cut sections, stain, wash well in distilled water, dehydrate for 3 minutes in absolute alcohol, "clear" in clove oil or xanthol and mount in C. balsam. Carmine is, perhaps, the best stain for fishes teeth. If it is used, however, it is necessary before transferring to distilled water to pass the sections quickly through weak acetic acid as this "fixes" the stain. If gold chloride is used the specimens must be mounted in glycerine jelly.

*It is not Necessary to Cut Sections of Enamel to Demonstrate the Prisms.* 1st. Soften the enamel by immersion in 10 per cent. solution of HCl remove by means of a needle point, or fine brush a small portion to a slide; put a drop of normal salt solution on the top of the enamel and press down the cover glass. Then run a solution of carmine or orange-rubine beneath the cover glass and draw off the excess with a little blotting paper. Wash the stain away further by irrigation with a weak HCl or $HC_2H_3O_2$ and mount in this solution or acidified glycerine after Beale's plan.

*Staining with Chloride of Gold.* No other stain marks out so clearly the minute anatomy of the soft tissues which penetrate bone and dentine, in fact, its excellence as selective stain would long ago have been demonstrated but for the recognized text-books speaking of the great difficulty of using it and the length of time in takes, and being only applicable to fresh tissues.

True, fresh tissues always stain faster, but teeth, and bone and indeed other tissues can be stained after having been severed from the living body for a long time, sometimes weeks. Avoid the use of metal instruments, bone, wood or quill being preferable. The use of steel does not, however, doom the staining to failure. To stain (a) wash the sections in a solution of bicarbonate of soda. (b) Put some 1 per cent. solution of gold chloride in a watch glass, test it with litmus paper, and if acid, neutralize with (c) bicarbonate of soda by drops ; place sections in the solution and cover the watch glass with some lid to keep it in total darkness for from ½ to 1 hour until the sections are straw-color.

Remove sections from staining fluid to distilled water, leave covered over (they must not be exposed to the light for more than a few seconds) for a few minutes.

(d) Put some 1 per cent. formic acid in a watch glass, float the glass on hot water, put the sections in the acid, cover them over and keep them in the dark and fairly hot until they turn crimson which is in about an hour. (e) When stained immerse the sections in cold distilled water for about ½ an hour. (f) Dry sections and mount them in glycerine jelly. Avoid C-Balsam. Keep gold chloride bottle in the dark. (16)

Sections to show pulp (particularly hyperæmia) are difficult to make and to retain the natural injection. 1st. Catch your hare *i. e.*, capture the condition—examine a suitable case when one is presented, note condition of the tooth itself, etc. Remember *the condition of the tooth at the moment of extraction, especially as to pain* as it is of vast importance in sudying this object. Extract the tooth; place in Muller; do not handle or disturb in any way for a week, at least. Then harden; wrap the tooth in muslin and place in the jaws of a powerful vice (not one where the jaws are so weak that they will spring together on cracking the tooth, as it will crush the pulp) and steadily close them until the tooth cracks open. If skilfully done the line of fracture will be the long axis. Then place in Muller freshly filtered and carefully lift the pulp from its cavity. (Carefully do this for the dentinal fibrils will be pulled out a considerable length). Now place in a thin solution of gum arabic to which add some gum camphor, salicylic, thymol, or carbolic acid to prevent mould. *In no case must this gum be strong enough to float the pulp.* If of greater specific gravity than the pulp the tissue shrinks. Evaporate the gum arabic solution slowly to the consistence of a thick jelly. This should require 3 or 4 days to thoroughly penetrate the pulp. When the solution is hard enough to handle, the pulp is taken up (c) some mucilage placed in the position for cutting on a piece of cork afloat on alcohol with the pulp side down. In from 12 to 36 hours the surface will be hardened from the abstraction of the $H_2O$ by alcohol. Don't let it get too hard. Invest in a microtome; use paraffin, or some suitable substance for imbedding and allow to stand 12 to 24 hours. Several sections can be cut if desired. The specimen being kept wet in alcohol all the time. Mount direct in glycerine without dissolving the mucilage, or dissolve it out in tepid distilled water stain the pulp with hæmatoxylin or fuchsin and mount in Canada Balsam.

If a tooth is extracted during a paroxysm of pain inflammation of the pulp is almost uniformly accompanied by the signs of hyperæmia, they being present in a marked degree in the immediate neighborhood of the inflammatory area; but if the tooth is extracted during a period of quiet, the hyperæmia is limited to the vessels within the inflamed area.

*Celloidin.* After hardening well place for 24 hours in equal parts of ether and alcohol, transferring to a syrupy solution of celloidin, made by dissolving celloidin in a mixture of equal parts of alcohol and ether. Leave it for about 24 hours; cover the object with a thicker solution of celloidin; it is allowed to remain in it for 24 hours. Embed when ready on cork. Spread on easily a little of the celloidin solution and allow to dry. Then another coat and let dry. Now place on it the specimen as quickly as possible before the celloidin begins to harden. Now cover the whole with successive layers of the celloidin solution until the object is built up quite firm. When it has dried remove the celloidin from the glass with a sharp knife, and if necessary trim the bed to a proper size and form.

*To Place on Cork.* Coat the cork with celloidin solution and let it dry, (to prevent air rising from the cork). The object is now placed in its hardened matrix and mixing cell, as on the cork, by means of celloidin. Let dry in air till it retains its shape well. Drop the cork into 50 per cent. alcohol and it can usually be cut after soaking it for one hour.

For dental embryological work it is excellent.

*Developing Teeth-Sections.* Take the teeth that are forming in the jaws of embryoes, at or nearly the time of birth, while the tissue is still warm if possible. Place in $\frac{1}{4}$ to $\frac{1}{2}$ of 1 per cent. solution of chromic acid and change daily for 3 or 4 days. The edges of the dentine that were calcified are found sufficiently softened to make a number of sections. Take the teeth from the acid solution, wash in distilled water and then place in a solution of gum arabic for several hours. Then put in a solution of alcohol to take out the water. Paraffin and wax are melted and poured into a convenient mold. When clouded with cooling, embed the tissue, and cut it until the calcified tissue is reached. Place the sections in distilled water for a few minutes to dissolve out the gum and then put in glycerine and alcohol and mount in glycerine.

For further details on Embryonic Teeth Sections see Paper on Histology of the Teeth in the *Jour. of Microscopy and Natural Science*, New Series, Vol. II, 1889, where they are given at length.

*Prepairing Sections of Decayed Dentine.* Select a freshly extracted decayed tooth, wash out all the particles of food and break away the margins of enamel so as to expose the softened dentine as much as possible. Then with a sharp instrument cut away the decayed portion from the sound dentine, keeping the instrument *well* to the latter and we thus get a large piece of decayed dentine. Immediately freeze the tissue in gum, stain and mount.

*Staining Tissues.* This requires a little practice to secure good results so we will take the simplest, namely, logwood. Buy a good sample or make one from the many receipts found in all histological text books and then filter 12 or 15 drops in a watch-glass, and add a few drops of distilled water, stir well to mix the agents. Place in a few sections (the fewer and the slower the stain the better) from the spirits and let them straighten out on the stain and then gently press under the logwood, leave it for about ten minutes (the time differs in every case) and then test them by washing in *tap water.* This is about the only time we have occasion to use any other than distilled water, but the former fixes the stain the best. When deep enough and well washed to remove all precipitates, place the sections in spirit for a good ten minutes to dehydrate and then in clove oil, and then mount in Canada Balsam or what other media is prepared. All the stains are used, with only slight modifications, in a similar manner.

*Picro-Carmine* is a very useful stain on account of its double-staining property. Place the sections in a strong solution for from 10 to 30 minutes, then wash in acidulated water (distilled water to which 1 or 2 drops of acetic or picric acid have been added). Leave in this for 15 to 30 minutes, wash quickly in alcohol and then transfer to clove oil and mount in balsam. Some histologists are against the use of balsam as a medium and advise glycerine or Farrant's media. Logwood is a good combining stain with the above. Fuchsin is also a good stain.

*Bibliography Useful to Dental Histologists.*

1. Gysi, Dental Caries Under the Microscope, *Dental Cosmos*, April, 1887.

2. Weil, Zur Histologie der Zahnpulpa, 1887.
3. Miller & Underwood, Trans. Odont. Soc. of Gt. Britain, 1884, p. 222.
4. Stricker's Histology, Vol. I.
5. Abbot, *Dental Cosmos.*
6. Weil, Zeitschrift. f. Wiss. Mikr V. (1888) pp. 200-2.
7. Mummery J. H., T. Odont. Gt. Britain, XXII (1890) p. 207.
8. Hopewell-Smith W. A. *J. Br. Dental Assoc.* XI 1890. pp. 310-2.
9. Brittan W. C. Dental Register, Vol. 41, 1887, p. 19,
10. Andrews, Dental Register, 1887, p. 564.
11. Flexner S. Dental Register, 1887, p. 336-341.
12. Latham V. A. *Scientific Enquirer*, Vol. I, 1887, pp, 196.
13. " " " *J. of Micro. and Natural Science N. S.* Vol. II, 1889.
14. " " " Dental Register, Oct. '88, pp. 477. 482.
15. Dental Cosmos, Register and American System of Dentistry.
16. Underwood A. S. *J. B. Dent. Assoc.* IX, 1890, pp. 696. 697.
17. Miller W. D. Micro-organisms of the Mouth.

Dr. Virchow Denounces Kochism.—The lower house of the Prussian Diet on May 9th voted 165,000 marks for Prof. Koch's institute. Professor Virchow opposed the grant and denounced Kochism, claiming that it had proved a failure. He warned the doctors who were using the lymph that they ran a great risk if they persisted in treating their patients with the alleged remedy.—*Med. Record.*

Four Years' Course at the Harvard Medical School.—The faculty of the Harvard Medical School, at their last meeting, voted that with the class entering in September, 1892, the regular course necessary to obtain the degree of M.D. shall be four years.—*Boston Med. and Surgical Journal.*

